# Satellite cells fail to contribute to muscle repair but are functional in Pompe disease (glycogenosis type II)

**DOI:** 10.1186/s40478-018-0609-y

**Published:** 2018-10-31

**Authors:** Lydie Lagalice, Julien Pichon, Eliot Gougeon, Salwa Soussi, Johan Deniaud, Mireille Ledevin, Virginie Maurier, Isabelle Leroux, Sylvie Durand, Carine Ciron, Francesca Franzoso, Laurence Dubreil, Thibaut Larcher, Karl Rouger, Marie-Anne Colle

**Affiliations:** 1 PAnTher, INRA, École Nationale Vétérinaire, Agro-alimentaire et de l’alimentation Nantes-Atlantique (Oniris), Université Bretagne Loire (UBL), Nantes, F-44307 France; 2 INSERM UMR1089, Université de Nantes, Centre Hospitalier Universitaire, Nantes, France; 3 BIA, INRA, Centre INRA Pays de la Loire, Nantes, F-44300 France

**Keywords:** Pompe disease, GAA KO 6^neo^/6^neo^ mice, Skeletal muscle, Satellite cell, Muscle regeneration, Lysosome, Glycogen overload, Splitting

## Abstract

Pompe disease, which is due to acid alpha-glucosidase deficiency, is characterized by skeletal muscle dysfunction attributed to the accumulation of glycogen-filled lysosomes and autophagic buildup. Despite the extensive tissue damages, a failure of satellite cell (SC) activation and lack of muscle regeneration have been reported in patients. However, the origin of this defective program is unknown. Additionally, whether these deficits occur gradually over the disease course is unclear. Using a longitudinal pathophysiological study of two muscles in a Pompe mouse model, here, we report that the enzymatic defect results in a premature saturating glycogen overload and a high number of enlarged lysosomes. The muscles gradually display profound remodeling as the number of autophagic vesicles, centronucleated fibers, and split fibers increases and larger fibers are lost. Only a few regenerated fibers were observed regardless of age, although the SC pool was preserved. Except for the early age, during which higher numbers of activated SCs and myoblasts were observed, no myogenic commitment was observed in response to the damage. Following in vivo injury, we established that muscle retains regenerative potential, demonstrating that the failure of SC participation in repair is related to an activation signal defect. Altogether, our findings provide new insight into the pathophysiology of Pompe disease and highlight that the activation signal defect of SCs compromises muscle repair, which could be related to the abnormal energetic supply following autophagic flux impairment.

## Introduction

Pompe disease (glycogen storage disease type II; OMIM 232300) is a lysosomal storage disorder due to a mutation in the gene encoding acid-alpha glucosidase (GAA), which is a unique hydrolase that enables glycogen breakdown into glucose in lysosomes [25, 27]. GAA deficiency causes the abnormal accumulation of lysosomal glycogen in many cell types, leading to cell and, subsequently, tissue dysfunction. Cardiac, respiratory and skeletal muscles are the most severely affected [79, 82]. Two forms of the disease are classically described, i.e., the infantile form, which is the most severe due to almost no residual GAA activity (< 2%), and the late-onset form, which presents a variable spectrum of residual GAA activity (2–40%) and is more progressive. In both forms of Pompe disease, patients exhibit a severe impairment of skeletal muscles that clinically manifests as muscle weakness [5, 31]. Histologically, skeletal muscle is characterized by vacuolized fibers resulting from the accumulation of enlarged glycogen-filled lysosomes and autophagic debris [21, 46, 63, 65, 77, 86]. Macro-autophagy (often referred to as autophagy) is a physiological pathway involving the degradation of cellular components through lysosomal machinery and energy generation during starvation [42]. A double-membrane vesicle called autophagosome sequestrates and delivers a part of the cytoplasm to lysosomes for degradation. In Pompe disease, massive autophagic buildup occurs due to an impaired fusion between autophagosomes and dysfunctional lysosomes [51, 55]. The accumulation of lysosomal and autophagic buildup has been reported to be responsible for the ultrastructural damage in myofibers by disrupting the proper positioning of contractile elements [12, 21, 56, 77]. Despite this extensive intracellular damage, skeletal muscles in Pompe patients intriguingly exhibit very few necrotic fibers subjected to macrophage-mediated phagocytosis and regenerative elements [63]. This feature defines an atypical tissue response as muscle damage classically triggers the activation of myogenic progenitors called satellite cells (SCs) located in a quiescent state between the sarcolemma and *basal lamina* of myofibers [41]. Consequently, these resident cells proliferate, enter the myogenic differentiation program and fuse to existing damaged fibers or each other for new myofiber formation [33, 62]. Recently, a potential dysfunction of SCs has been suggested in Pompe disease. Indeed, SCs in muscle biopsies from patients at different ages with different disease severities were shown to not differ from those from controls in terms of proliferation and differentiation [63]. The hypotheses proposed to explain this failure of SC activation include the absence of the activation signal, the presence of an inhibitory factor or impaired autophagy.

To deeply investigate the muscle regenerative process and particularly the defective activation of SCs over the Pompe disease course, for the first time, we performed an extensive longitudinal histological follow-up using a GAA-KO 6^neo^/6^neo^ mouse model [54]. This model lacks enzyme activity and displays a glycogen accumulation profile similar to that described in patients. Additionally, this model recapitulates the critical features of both forms of the disease, including muscle pathology and weakness. Many works have reported that similar to patients, lysosomal accumulation and autophagic buildup in the skeletal muscle of Pompe mouse models [37, 56] interrupt the cross striation of the contractile apparatus in fibers [16, 54, 69]. Except for demonstrations of the large number of centronucleated fibers in muscle from aged Gaa^−/−^ mice suggesting that degenerative and regenerative events have occurred [14, 88, 89], studies investigating the myopathic abnormalities occurring with the progression of the disease have rarely been performed. Therefore, the understanding of the skeletal muscle pathology is incomplete.

In the present work, we considered two skeletal muscles from anterior and posterior limbs and four ages ranging from 1.5 to 9 months and compared Gaa^−/−^ mice to their wild-type (WT) littermates. Specifically, we histologically investigated the glycogen overload and cytoplasmic accumulation in both lysosome and autophagic vesicles over the course of the disease. We combined these data with biochemical and infrared microspectroscopy approaches. Furthermore, we performed an extensive quantitative immunohistochemical analysis of the myopathic abnormalities, the regeneration process and particularly the SC behavior to obtain a more in-depth knowledge regarding the skeletal muscle pathophysiology in the Gaa^−/−^ mice. Finally, we completed this investigation by inducing muscle injury in vivo to explore the response of SCs from Gaa^−/−^ mice to acute tissue stimulation.

## Materials and methods

### Study design

This was a longitudinal pathophysiological study designed to define the histological differences among experimental groups and to investigate the behavioral features of SCs faced to an injury (Gaa^−/−^ mice muscles vs. WT mice muscles; Cardiotoxin [CTX; Latoxan, Portes-lès-Valence, France]-injured Gaa^−/−^ mice muscles vs. CTX-injured WT mice muscles). The disease impact was assessed in situ by histomorphological, biochemical, infrared microspectroscopy and in vivo testing. Four endpoints were selected to sample and analyze the course of the disease: a short-term (1.5 month [mo]), two mid-term (4 and 6 mo) and an advanced term (9 mo). A first cohort included four groups of both Gaa^−/−^ and WT male mice (*n* = 5 per time-point). The second cohort included 4-mo-old Gaa^−/−^ and WT male mice that received an intra-muscular CTX injection to induce acute injury. The mice were sacrificed 4, 7 and 21 days post-injection (dpi; *n* = 4 Gaa^−/−^ and WT mice per time-point).

### Animals

The Gaa^−/−^ mice (GAA-KO 6^neo^/6^neo^ mouse model) used in this study have a targeted disruption of exon 6 in the gene encoding GAA and, consequently, lack enzymatic activity [54]. The breeding heterozygous 6^neo^/6^neo^ mice were kindly provided by Dr. Nina Raben (NIH, Bethesda, MD, USA) and kept in the specific pathogen-free animal facility at Oniris (Nantes-Atlantic National College of Veterinary Medicine, Food Science and Engineering, Nantes, France). Gaa^−/−^ homozygous breeding was performed, and WT littermates were bred as controls. The mice were provided food (standard mouse chow) and water ad libitum and housed under a 12:12 h dark:light cycle at 22 °C. All procedures were performed in accordance with the guidelines of the European Council for the Care and Use of Laboratory Animals. The study was approved by the Ethics Committee on Animal Experimentation of the Pays de la Loire region, France (authorization number APAFiS #1267).

### Histological and immunofluorescence analyses

The *Tibialis anterior* (TA) and *Triceps brachii* (TB) muscles were dissected, positioned on tragacanth gum, snap-frozen in isopentane prechilled in liquid nitrogen and stored at − 80 °C until sectioning. Transverse muscle sections (10-μm thickness) were stained with hematoxylin-eosin-saffron (HES) or periodic-acid-Schiff (PAS). The microscope images were viewed under a Nikon Eclipse 90i**®** microscope (Nikon France, Champigny sur Marne) using NIS-Elements software (Nikon, Champigny sur Marne, France). To study the CTX-injected muscles, four transverse frozen sections (10-μm thickness, each separated by 100 μm intervals) were considered to ensure that the entire injected area was included. The muscle sections were blindly analyzed by three veterinary pathologists certified by the European College of Veterinary Pathologists (TL, FF and MAC).

For the immunofluorescence studies, the frozen sections were first permeabilized (30 min, room temperature [RT]) using 0.1% Triton X-100 (Sigma-Aldrich, Saint Quentin-Fallavier, France) and incubated (30 min, RT) in blocking buffer (mixture of 5% goat serum and 5% bovine serum albumin [BSA] in 0.1 M phosphate-buffered saline [PBS], Sigma-Aldrich). Then, the sections were incubated (overnight at 4 °C) with the following primary antibodies: rabbit polyclonal anti-LC3B (1:100, L7543, Sigma, Saint Quentin-Fallavier, France), anti-laminin (1:800, L9393, Sigma), anti-collagen I (1:100, PA1–85319, Invitrogen), and anti-Ki67 (1:500, ab15580, Abcam, Cambridge, UK) antibodies; mouse monoclonal IgG1 anti-dystrophin (1:50, NCL-DYS2, Novocastra Laboratories, Newcastle on Tyne, UK), anti-developmental isoform of myosin heavy chain (1:10, NCL-MHCd, Novocastra), anti-Pax7 (1:10, PAX7, Developmental Studies Hybridoma Bank/DSHB, Iowa City, IA, USA), anti-MyoD (1:25, M3512, Dako, Glostrup, Denmark) and anti-Myogenin (1:25, F5D, DSHB) antibodies; and rat monoclonal anti-LAMP1 (1:50, 553792, BD Pharmingen, San Jose, CA, USA) and anti-F4/80 (1:200, MCA 497GA, Bio-Rad AbD Serotec Oxford, UK) antibodies. The sections were washed with 0.1 M PBS and then incubated with Alexa**®** red 555 or green 488 secondary antibodies (1:300, A11008; A21127; A21434, Invitrogen, Carlsbad, CA, USA) for 1 h at RT. The nuclei were counterstained with DRAQ5 (DR50200, Biostatus, Loughborough, UK). The acquisitions were performed under a confocal laser microscope (LSM 780, Zeiss, Oberkochen, Germany) using ZenBlack software (Zeiss, Oberkochen, Germany).

### Glycogen storage quantification

For the biochemical analysis, the TA and TB muscles were rapidly dissected, frozen in liquid nitrogen and stored at − 80 °C until processing. The tissues were homogenized in phosphate buffer containing protease inhibitors (Roche, Mannheim, Germany) using Precellys® (Ozyme, Montigny Le Bretonneux, France). The homogenates were centrifuged at 13,000 rpm for 10 min at 4 °C, and the resulting supernatant was used for the biochemical measurement of the glycogen content as described elsewhere [28]. Briefly, the tissue extracts were boiled for 3 min and incubated at 54 °C for 1 h with or without 5 U/mL Aspergillus niger amylo-α-1,4-α-1,6 glucosidase (Roche, Mannheim, Germany), which converts glycogen to glucose. The samples were centrifuged, and the glucose level in the supernatant was determined using an AmplexRed® Glucose Assay Kit (A22189, Invitrogen, Cergy-Pontoise, France) per the manufacturer’s instructions.

### Histomorphometry

The microscopic fields were randomly selected to evaluate at least 1,000 muscle fibers. The quantifications and morphometric analyses of the immunolabeled sections were blindly performed using 20× magnification. Fiji freeware (https://fiji.sc/) was used for the cell counting and area measurement. To assess the autophagic buildup in the Gaa^−/−^ mouse muscles, the proportion of fibers containing LC3^+^-aggregates relative to the total number of muscle fibers in the field (delimited by dystrophin immunolabeling) was determined in each section. The surface occupied by these LC3^+^-aggregates was expressed as a ratio of the total fiber area. To characterize macrophage infiltration, the mean number of macrophages per muscle fiber in each field was counted using immunolabeling specific to the cytoplasmic marker F4/80. In the CTX-injected muscles, macrophage infiltration was expressed as the percentage of the muscle section area occupied by F4/80 immunolabeling since the vast infiltration did not allow for counting each cell separately. For that, the same threshold was applied to each picture using Fiji software. To investigate anisocytosis, the minimum Feret diameter (MinFeret; shortest distance between two parallel tangents of the muscle fiber edges) of the dystrophin-immunolabeled muscle fibers was used and determined semi-automatically. The recognition of the muscle fiber perimeter was manually controlled to prevent potential errors. The regenerative activity was measured by counting the number of fibers expressing the developmental isoform of the myosin heavy chain (dMyHC) relative to the total number of muscle fibers in the field. The number of muscle fibers that appeared split into two or more fragments was expressed relative to the total number of muscle fibers detected on the HES-stained sections. To quantify fibrosis, the area occupied by collagen I labeling in the entire field was automatically measured using a determined threshold in Fiji freeware.

### Infrared microspectroscopy

Transverse sections (10-μm thickness) of TA muscle from 1.5- and 9-mo-old mice (*n* = 3 Gaa^−/−^ and WT mice per age) were placed on Zinc Sulfate windows (Crystran Limited, Poole, UK). An infrared (IR) spectrometer Tensor 27 coupled to a Hyperion 2000 microscope (Bruker, Billerica, MA, USA) was used to collect spectra in the range of 4000–700 cm^− 1^ at a spectral resolution of 8 cm^− 1^ and a spatial resolution of 20 μm × 20 μm. In total, 100 muscle fibers were selected from each sample, and an average of 300 scans per fiber resulted in a collection of 100 spectra per sample. Using Unscrambler® software, the spectral data were first baseline corrected and unit vector normalized. The second derivatives of the spectral data were assessed (9-point Savitzky-Golay filter) to enhance the spectral resolution of the absorption bands. The second derivative IR spectra were analyzed by applying a principal component analysis (PCA). The computation of the principal components was based on the non-linear iterative projections by the alternating least-squares (NIPALS) algorithm. While the score plots allowed for a comparison of the IR spectra, the corresponding loading plots revealed the main characteristic absorption bands. The three major peaks at 1180–950 cm^− 1^, 1770–1720 cm^− 1^ and 3100–2800 cm^− 1^ were assigned to glycogen, esters and lipids, respectively, according to the literature [43]. The comparisons among the samples were performed by using OPUS® software to measure the area under each assigned peak with normalization against the protein amides peak area (1720–1480 cm^− 1^).

### Skeletal muscle injury

The TA muscles from the Gaa^−/−^ and WT mice were injected with 30 μl of 12 μM CTX in saline in the midbelly portion of the muscle as described elsewhere [19]. All procedures were conducted under anesthesia using an intra-peritoneal injection of a solution containing 100 mg/kg Ketamine and 10 mg/kg Xylazine (Merial, Lyon, France). For the post-operative analgesia, the mice received a subcutaneous injection of 50 μg/kg buprenorphine (Buprecare, Axience, Pantin, France). The animals were sacrificed on days 4, 7 and 21 following the CTX-induced injury (*n* = 4 Gaa^−/−^ and WT mice per time-point) for the longitudinal assessment of muscle degeneration and regeneration.

### Statistical analysis

The data are expressed as the average ± standard error of the mean (SEM). One-way analysis of variance (ANOVA) was performed, followed by a Sidak multiple comparison post hoc test as appropriate, to reveal the influence of the age of the Gaa^−/−^ mice on the variables of interest. To compare the impact of both the status (Gaa^−/−^ versus WT) and age (1.5, 4, 6 and 9 mo) of the animals on the variables of interest, a two-way ANOVA was used, followed by the application of Sidak multiple comparison post hoc tests to both factors. The Sidak test is designed to compensate for the inflation of first-type risks, which are well-known phenomena occurring in multiple comparison procedures. These statistical analyses were performed using GraphPad Prism v.6.0 (GraphPad Software, La Jolla, CA, USA). A *p*-value of 0.05 or less was considered significant.

## Results

### GAA defect prematurely results in a saturating glycogen overload in skeletal muscles

To characterize the skeletal muscle pathology in Pompe disease, a longitudinal study was performed on a forelimb and a hindlimb muscle, i.e., the TA and the TB muscles, respectively, in a GAA-KO 6^neo^/6^neo^ mouse model compared to those in WT littermates. Furthermore, four ages corresponding to 1.5, 4, 6 and 9 mo were considered.

**Fig. 1:**
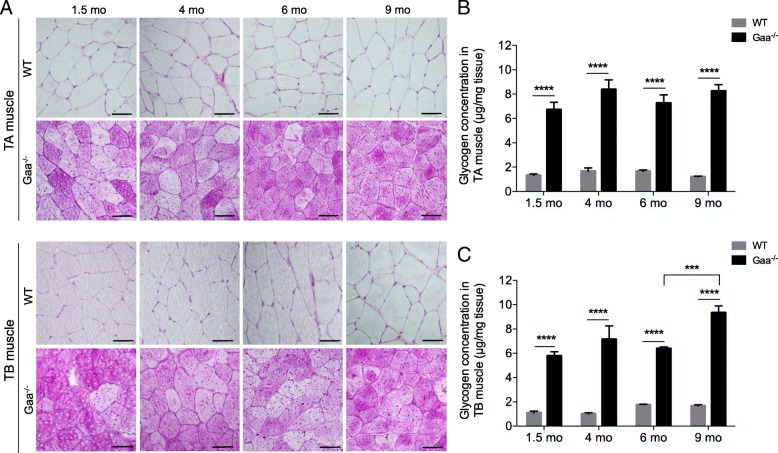
Kinetics of glycogen accumulation in skeletal muscles from Gaa^−/−^ mice. **a**: Periodic-Acid Schiff (PAS) staining of *Tibialis anterior* (TA) and *Triceps brachii* (TB) muscle sections from Gaa^−/−^ mice and WT littermates at 1.5, 4, 6 and 9 mo of age. **b**, **c**: Glycogen concentration measurement in tissue extracts from TA and TB muscles from Gaa^−/−^ and WT mice. Scale bars = 50 μm. Statistics: Two-way ANOVA with Sidak post hoc test; *n* = 5 animals per group; ***p < 0.001; ****p < 0.0001

We determined that the TA muscle cross-sections from the 1.5-mo-old Gaa^−/−^ mice exhibited a diffuse purple cytoplasmic pattern with the PAS staining (Fig. 1a). Variable intensities were observed among the fibers, which all clearly appeared to be PAS^+^. The presence of intense darker spots that may correspond to glycogen-filled lysosomes was also observed in the cytoplasm of the muscle fibers. A similar finding was observed in the muscles from the 4-, 6- and 9 mo-old Gaa^−/−^ mice*.* In comparison, the PAS-stained sections of the TA muscle from the WT mice showed that the muscle fibers had a uniform pale cytoplasm regardless of the age, highlighting the lack of glycogen accumulation. The same findings were observed in the TB muscle. The biochemical measurement confirmed these histochemical results, further revealing that the glycogen content in the TA muscle ranged from 6.72 ± 0.53 μg per mg of muscle tissue to 8.37 ± 0.80 μg/mg in Gaa^−/−^ mice aged from 1.5 to 9 mo (Fig. 1b). In comparison, the glycogen content remained constant in WT mice in this age range with an average of 1.46 ± 0.12 μg glycogen per mg of muscle tissue. This finding indicated that the glycogen content in the Gaa^−/−^ mice at each age was approximately 4-fold higher than that in the WT mice (*p* < 0.0001). Similar results were obtained in the TB muscle. The glycogen content ranged from 5.78 ± 0.34 μg/mg to 9.34 ± 0.57 μg/mg in Gaa^−/−^ mice aged from 1.5 to 9 mo (Fig. 1c). This finding revealed a slight but significant increase at the later time point (*p* < 0.001). In comparison, the glycogen content was 1.38 ± 0.19 μg per mg of muscle tissue in the 1.5- to 9-mo-old WT mice. Considering the four time-points, the glycogen content in the Gaa^−/−^ mice was approximately 3- to 4-fold higher than that in the WT mice (*p* < 0.0001). The glycogen content did not significantly differ between the TA and TB muscles from the Gaa^−/−^ mice at the different ages considered.

**Fig. 2:**
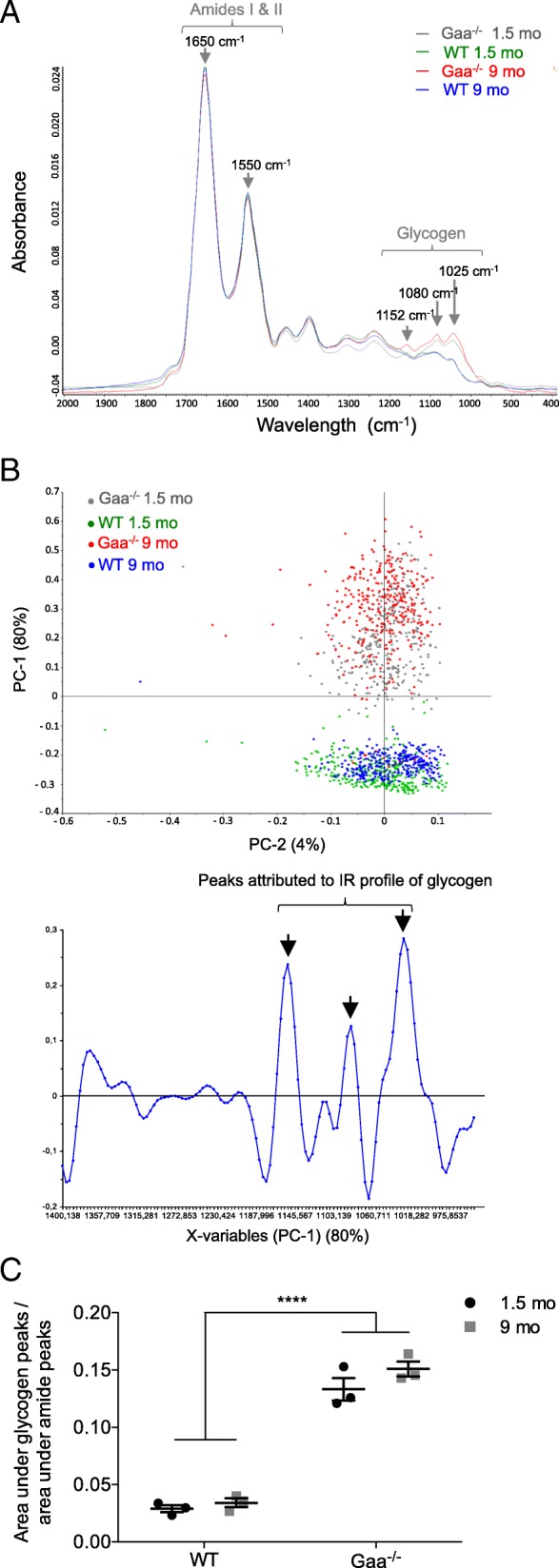
Glycogen accumulation in muscle fibers using infrared (IR) microspectroscopy. **a**: Representative raw IR spectra obtained from *Tibialis anterior* (TA) muscle fibers from Gaa^−/−^ and WT mice aged 1.5 and 9 mo (spectral range: 2000–800 cm^− 1^). **b**: Principal component analysis (PCA) of IR spectra in the 1400–950 cm^− 1^ range collected by mapping muscle fibers from Gaa^−/−^ and WT mice and corresponding loading plot (*n* = 100 spectra per mouse). **c**: Surface area under the curve of the glycogen peaks (spectral range: 1180–950 cm^− 1^) normalized against the protein peak area (spectral range: 1720–1480 cm^− 1^). Statistics: Two-way ANOVA with Sidak post hoc test; *n* = 3 animals per group; ****p < 0.0001

To complete the data obtained from whole muscle tissue, glycogen accumulation was investigated at the scale of the single fiber using free-labeled TA muscle sections from 1.5- and 9-mo-old Gaa^−/−^ and WT mice using the infrared IR microspectroscopy approach. As shown in Fig. 2a, the bands assigned to the carbohydrates of glycogen (IR absorption bands 1152, 1080 and 1025 cm^− 1^) were elevated in the Gaa^−/−^ mice in a comparison of the IR spectra obtained from muscle fibers from Gaa^−/−^ and WT mice at each age considered. A multivariate analysis was implemented to identify the possible trends in the changes observed in the glycogen spectral data set. A PCA was performed on the second-derivative spectra calculated from all spectra acquired considering the spectral region 1400 cm^− 1^ to 950 cm^− 1^ (Fig. 2b). The score plot revealed the following two independent clusters using the first two components, i.e., PC1 and PC2, which represent 80% and 4%, respectively, of the total variance: the first cluster grouped the Gaa^−/−^ mice, and the second cluster grouped the WT mice without clearly separating the old and young mice in each group. A closer examination of the corresponding loading plots of PC-1 (Fig. 2b, bottom) revealed that the main bands contributing to the cluster formation were the three bands assigned to glycogen at 1152 cm^− 1^, 1080 cm^− 1^ and 1025 cm^− 1^. In addition, these results were confirmed by the data obtained from the surface measurements performed using IR spectra area assigned to glycogen (Fig. 2c). Indeed, the surface measured under the glycogen peaks was 3-fold more important for the muscle fibers from the Gaa^−/−^ muscle fibers than for those from the WT mice. No differences in the glycogen peak area were observed between the 1.5- and 9-mo-old Gaa^−/−^ mice.

Overall, the results indicated that the GAA defect was associated with a glycogen overload that is present from the early stage of the disease at a saturating rate. This rate did not evolve over the course of the disease, even though a slight increase was observed at 9 mo in the TB muscle.

### Progressive cytoplasmic accumulation of LC3^+^_−_aggregates and enlarged lysosomes characterize Gaa^−/−^ mouse muscle

**Fig. 3:**
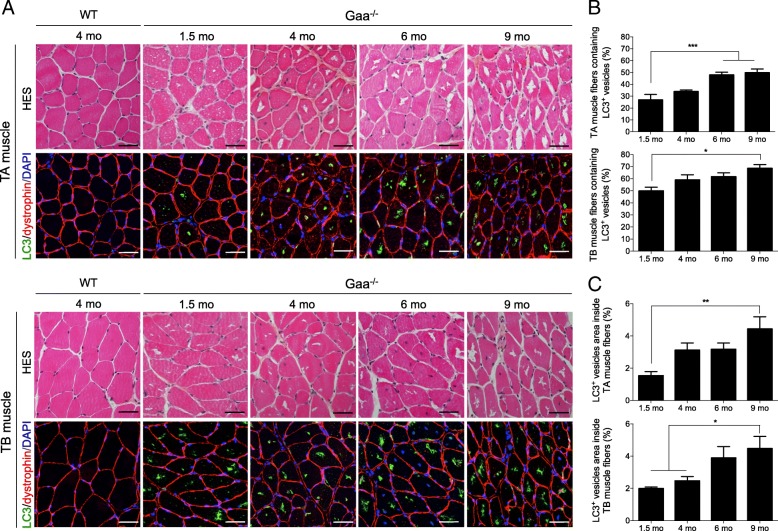
Structural abnormalities in skeletal muscle from Gaa^−/−^ mice and autophagic buildup over the disease course. **a**: Serial cross-sections of *Tibialis anterior* (TA) and *Triceps brachii* (TB) muscles stained with Hematoxylin-Eosin-Saffron (HES) and immunolabeled with anti-LC3 (green) and anti-dystrophin (red) antibodies. Nuclei are counterstained with DRAQ5 (blue). **b**: Quantification of autophagic buildup in fibers from TA and TB muscles. **c**: Quantification of the size of the LC3^+^-aggregates in TA and TB muscle fibers. Scale bars = 50 μm. Statistics: One-way ANOVA with Sidak post hoc test; n = 5 animals per group; *p < 0.05; **p < 0.01; ***p < 0.001

**Fig. 4:**
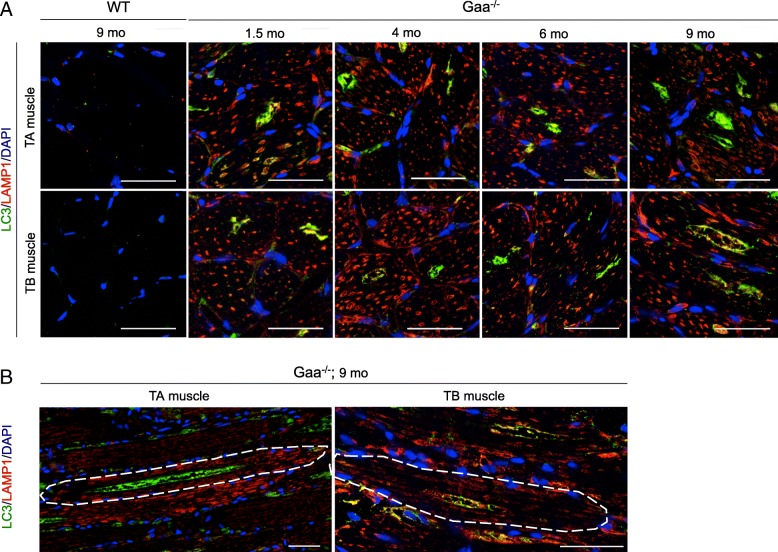
Autophagic vacuole and enlarged lysosome accumulation in skeletal muscles from Gaa^−/−^ mice. **a**: Cross-sections of *Tibialis anterior* (TA) and *Triceps brachii* (TB) muscles from Gaa^−/−^ mice at 1.5, 4, 6 and 9 mo of age and 9-mo-old WT mice subjected to LC3 (green) and LAMP1 (red) immunolabeling. Nuclei are counterstained with DRAQ5 (blue). **b**: Longitudinal sections of TA and TB muscles from 9-mo-old Gaa^−/−^ mice following immunolabeling with anti-LC3 (green) and anti-LAMP1 (red) antibodies. Nuclei are counterstained with DRAQ5 (blue). Scale bars = 50 μm

The TA and TB muscles from the Gaa^−/−^ mice displayed progressive and profound tissue remodeling beginning at 1.5 mo of age; in contrast, the muscles from the WT mice showed a typical tissue organization at the different time-points characterized by the presence of fibers exhibiting a regular shape and size and a homogenous eosinophilic cytoplasm in HES-stained cross-sections (Fig. 3a). In the Gaa^−/−^ mouse muscles, the intracytoplasmic vacuoles that appeared either optically empty or filled with rough content were first detected in the core of the fibers throughout the muscle section. While most vacuoles were positive for microtubule-associated protein 1 light chain 3 (LC3), which is classically defined as a membrane marker of autophagosomes, LC3^+^-aggregates were not visible in the vacuoles in the WT mice (Fig. 3a). The proportion of fibers containing LC3^+^-aggregates gradually increased from 26.84 ± 4.24% to 49.77 ± 3.16% in the TA muscles of the Gaa^−/−^ mice between 1.5 and 9 mo of age (Fig. 3b). In comparison, this proportion similarly evolved from 49.76 ± 3.24% to 68.48 ± 3.32% in the TB muscles from the corresponding mice, revealing that this muscle was more affected than the TA muscle at 9 mo of age (*p* < 0.01). The number of fibers containing LC3^+^-aggregates was 1.9-fold and 1.4-fold higher in the TA and TB muscles from the 9-mo-old mice than that in the 1.5-mo-old mice, respectively, revealing a progressive autophagic buildup (*p* < 0.001 and *p* < 0.05, respectively). As shown in Fig. 3c, we found that the size of the LC3^+^-aggregates progressively increased over the course of the disease and was 2.90-fold and 2.25-fold higher in the TA and TB muscles from the 9-mo-old mice than that in the 1.5-mo-old mice, respectively (p < 0.01). In addition, we established that both the TA and TB muscles from the Gaa^−/−^ mice were characterized by the presence of numerous abnormally enlarged lysosomes in the cytoplasm of all fibers from the 1.5-mo-old mice based on immunolabeling specific to lysosomal associated membrane protein 1 (LAMP1) (Fig. 4a). In comparison, LAMP1^+^ vesicles could not be observed in the WT mouse muscle probably due to its small size. The longitudinal sections of the TA and TB muscles from the 9-mo-old Gaa^−/−^ mice showed that the enlarged lysosomes were homogeneously distributed throughout the whole surface of the cytoplasm, surrounding the centrally located LC3^+^-aggregates aligned in the depth of the fibers (Fig. 4b). Altogether, these results showed that the skeletal muscles from Gaa^−/−^ mice aged 1.5 mo displayed a high number of enlarged lysosomes in the cytoplasm and progressively accumulated LC3^+^-aggregates, while the intensity in the TB muscle was more pronounced than that in the TA muscle. These aggregates increased in size, occupied up to 25% of the fiber area and affected more than 50% of the fibers at 9 mo of age.

**Fig. 5:**
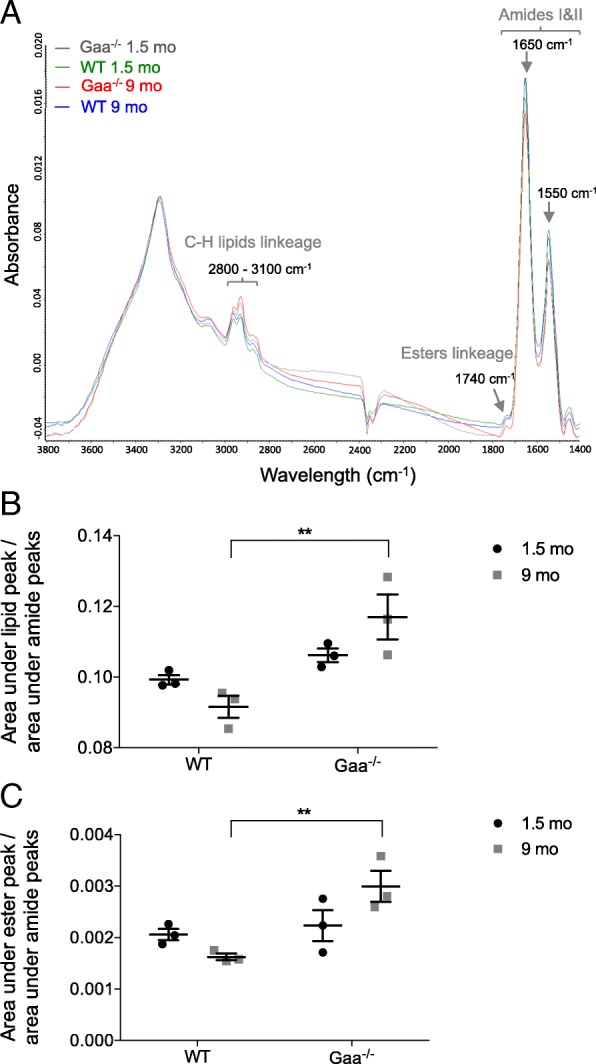
Lipid accumulation in muscle fibers from Gaa^−/−^ and WT mice using infrared (IR) microspectroscopy. **a**: Representative raw IR spectra obtained from *Tibialis anterior* (TA) muscle fibers from Gaa^−/−^ and WT mice at 1.5 and 9 mo of age (spectral range: 3800–1400 cm^− 1^). **b**, **c**: Surface area under the curve of the ester peak and the peak corresponding to the C-H linkage of lipids (spectral ranges: 1770–1720 cm^− 1^ and 3100–2800 cm^− 1^, respectively) normalized against the protein peak area (spectral range: 1720–1480 cm^− 1^). Statistics: Two-way ANOVA with Sidak post hoc test; n = 3 animals per group; **p < 0.01

Changes were also observed in the IR lipid spectral data set between the Gaa^−/−^ and WT mouse muscle fibers considering the CH linkage spectral regions (3100–2800 cm^− 1^) and the band assigned to the esters linkage (1740 cm^− 1^; Fig. 5a). Compared to the IR spectra of aged WT mice, the IR spectra obtained from the muscle fibers of the 9-mo-old Gaa^−/−^ mice were characterized by an elevation in the surface area of the peak assigned to the C-H linkage of lipids (3100–2800 cm^− 1^) and the surface area of the peak assigned to the esters linkage (1720–1770 cm^− 1^; Fig. 5b). These changes in the IR spectra of the Gaa^−/−^ mouse muscle, which for the first time are attributed to a specific IR microspectroscopy signature in the spectral range of lipids, could be related to the lysosome and autophagosome accumulation in the muscle fibers. Indeed, the membranes of these organelles are composed of phospholipids and cholesterol [3], and studies have previously used the IR vibrations of lipids and ester linkages to detect the accumulation of phospholipids and cholesterol in skeletal muscle membranes [71].

### Gaa^−/−^ mouse muscles do not exhibit typical degenerative lesions

**Fig. 6:**
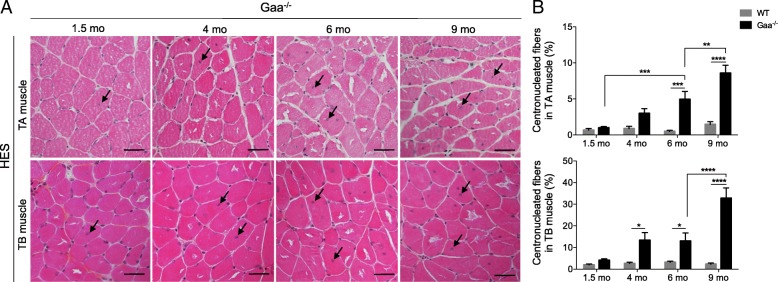
Presence of centronucleated fibers in skeletal muscle from Gaa^−/−^ mice. **a**: Hematoxylin-Eosin-Saffron (HES) staining of *Tibialis anterior* (TA) and *Triceps brachii* (TB) muscle cross-sections. Arrows indicate muscle fibers with one or more centrally located nuclei. **b**: Quantification of the centronucleation in TA and TB muscles. Scale bars = 50 μm. Statistics: Two-way ANOVA with Sidak post hoc test; n = 5 animals per group; *p < 0.05; **p < 0.01; ***p < 0.001; ****p < 0.0001

**Fig. 7:**
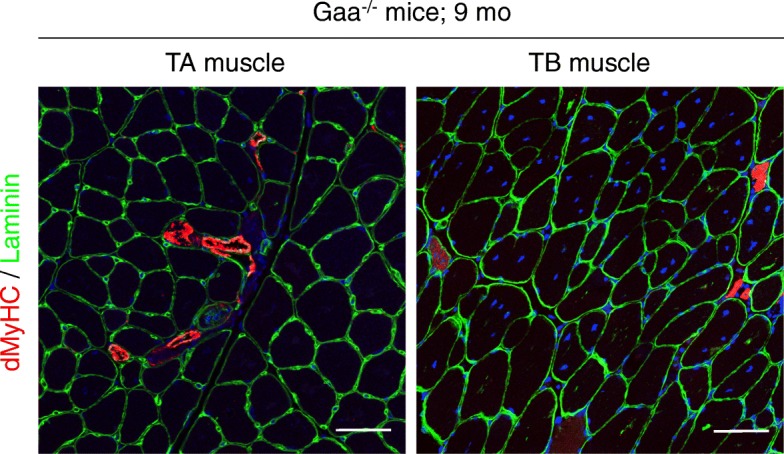
Detection of newly regenerated fibers in skeletal muscles from 9-mo-old Gaa^−/−^ mice. In transverse sections of *Tibialis anterior* (TA) and *Triceps brachii* (TB) muscles, immunolabeling of developmental myosin heavy chain isoform (dMyHC, red) and laminin (green) was performed. Scale bars = 50 μm

The histological analysis of the muscle cross-sections collected at the four time-points showed that both the TA and TB muscles from the Gaa^−/−^ mice intriguingly preserved a global tissue organization with a clearly defined fascicle and fibers separated by a thick and regular endomysium (Fig. 3a). However, the muscles were composed of fibers with an irregular shape and displayed some degree of heterogeneity. A diffuse and severely thickened endomysium was observed over the course of the disease in the TA muscle, whereas the endomysium thickening was focal and mild in the TB muscle at 9 mo of age. No adipose infiltration was observed. Interestingly, despite the presence of hyaline fibers, necrotic fibers were extremely rare in both skeletal muscles from the Gaa^−/−^ mice, and a maximum of 2 and 5 isolated fibers were observed in the entire muscle section at 9 mo of age. The TA and TB muscles from the Gaa^−/−^ mice were also defined by mild and diffuse macrophage (F4/80^+^ cells) infiltration that remained stable regardless of the stage of the disease and corresponded to a limited number of cells (0.21 ± 0.08% and 0.23 ± 0.07% F4/80^+^ cells/fiber compared to 0.06 ± 0.03% and 0.09 ± 0.04% in the TA and TB muscles from the WT mice, respectively). Overall, these data revealed that the biochemical and structural abnormalities observed in the skeletal muscles from the Gaa^−/−^ mice were not associated with a marked degenerative process. Intriguingly, some nuclei adopted a non subsarcolemmal location in the fibers from the Gaa^−/−^ mouse muscles, which is commonly considered as a regeneration marker (Fig. 6a). The nuclei mainly correspond to internalized nuclei with a random position in the cytoplasm of muscle fibers but also could appear as centrally located ones. In contrast, the conventional subsarcolemmal position was observed in almost all WT mouse fibers. The proportion of fibers exhibiting centrally located nuclei gradually increased in the TA muscle from the Gaa^−/−^ mice with an 8.74-fold increase between 1.5 and 9 mo of age (Fig. 6b). This finding contrasted with the constant percentage observed in the WT mice (ranging from 0.49 ± 0.14 to 1.47 ± 0.38 considering the different time-points). Compared to the corresponding WT mice, a higher proportion of centronucleated fibers was revealed in the TA muscle from the 6- and 9-mo-old Gaa^−/−^ mice (*p* < 0.001). Similar results were obtained in the TB muscle, including an 8.00-fold increase in non subsarcolemmal nuclei between 1.5 and 9 mo of age and a higher proportion of centronucleated fibers from the age of 4 mo (*p* < 0.05). At 9 mo of age, the proportion of centronucleated fibers was statistically more important for the TB muscle than the TA muscle, representing 32.75 ± 5.36% and 8.57 ± 1.25%, respectively (*p* < 0.0001). In both muscles, an increased proportion of fibers containing two or three central nuclei was observed over the course of the disease (20.4 ± 2.9% and 33.9 ± 2.7% compared to 4.4 ± 4.4% and 3.7 ± 3.7% in the TA and TB muscles at 9 vs. 1.5 mo of age, respectively). A maximum of 5 nuclei were found in some rare TB muscle fibers. The muscle regenerative activity was assessed using immunolabeling specific to the dMyHC isoform, whose expression is restricted to developmental and regeneration processes. As illustrated in Fig. 7, only a few dMyHC^+^ small-sized myoblasts were observed in both the TA and TB muscles regardless of the time-point considered, while none were found in the WT mouse muscles. This finding indicated that the increasing number of cytoplasmic nuclei positioned randomly observed over the course of the disease was not strictly correlated to the formation of newly regenerated fibers in the Gaa^−/−^ mouse muscle.

**Fig. 8:**
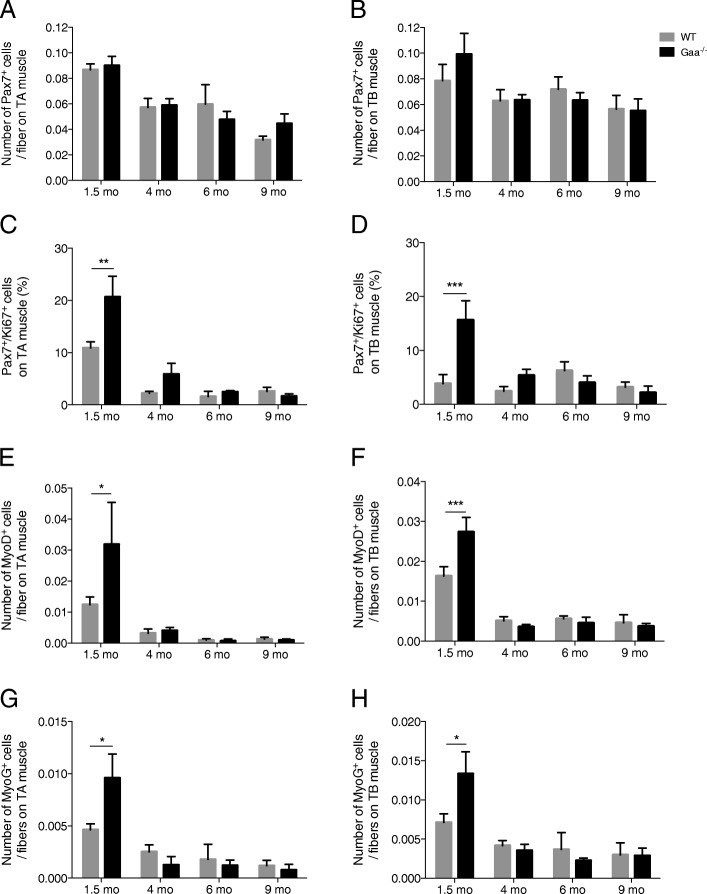
Pool, activation state and commitment level of muscle satellite cells from Gaa^−/−^ and WT mice.. **a**, **b**: Number of Pax7^+^ cells in *Tibialis anterior* (TA) and *Triceps brachii* (TB) muscles at 1.5, 4, 6 and 9 mo of age. **c**, **d**: Proportion of activated (Ki67^+^) satellite cells in TA and TB muscles. **e**, **f**: Number of MyoD^+^ cells per fiber in TA and TB muscles. G, H: Number of MyoG^+^ cells per fiber in TA and TB muscles. Statistics: Two-way ANOVA with Sidak post hoc test; n = 5 animals per group; **p < 0.01; ***p < 0.001

To better understand the regeneration process in Pompe disease, we first investigated the behavior of SCs since their contribution is required for the repair of damaged muscle fibers. We demonstrated that the number of Pax7^+^ cells located beneath the surrounding *basal lamina* and outside the myofiber plasma membrane, which defines the SC pool, was 0.090 ± 0.008 cells per fiber in the TA muscle from the 1.5-mo old Gaa^−/−^ mice compared to 0.087 ± 0.005 cells per fiber in that from the WT mice (Fig. 8a). Between 4 and 9 mo of age, this number decreased in the Gaa^−/−^ mice and always remained below 0.07 cells per fiber, which was similar to that in the WT mice. The same results were obtained in the TB muscle (Fig. 8b). Altogether, these results showed that the SC pool was preserved in the Gaa^−/−^ mice and that the lack of regeneration could not be attributed to its exhaustion. Then, the activation state of the SCs was analyzed by determining the number of Pax7^+^ cells that express the cell proliferation marker Ki67. At 1.5 mo of age, the proportion of Pax7^+^/Ki67^+^ cells was 20.65 ± 4.00% in the TA muscle from Gaa^−/−^ mice, while that in the WT mice was 10.90 ± 1.19% (Fig. 8c). Concerning the TB muscle in the Gaa^−/−^ and WT mice, this proportion was 15.67 ± 3.54% and 3.85 ± 1.65%, respectively (Fig. 8d). Thus, the number of Pax7^+^/Ki67^+^ cells was 1.89-fold and 4.07-fold higher in the TA and TB muscles from the Gaa^−/−^ mice than those from the WT mice, respectively (*p* < 0.01 in the TA muscle and *p* < 0.001 in the TB muscle). Furthermore, this number was similar between the Gaa^−/−^ and WT mice from the age of 4 mo, revealing that the TA and TB muscles in the Gaa^−/−^ mice had the same proportion of activated SCs as those in the WT mice. In addition, we determined that compared with the WT mice, the 1.5-mo-old Gaa^−/−^ mouse muscles showed a higher number of nuclei positive for the myogenic regulatory factors Myod and Myogenin (MyoG; Fig. 8e-h). No differences were observed between the Gaa^−/−^ and WT mice from 4 mo of age with a low (< 1%) number of Myod^+^ and MyoG^+^ nuclei per muscle section of the TA and TB muscles. Altogether, these data importantly revealed that the pool of SCs was unaltered over the course of Pompe disease but that its activity is strictly increased at the early stage. Indeed, no commitments into the myogenic lineage were observed from 4 mo of age in the Gaa^−/−^ mice, while an increase in structural abnormalities was observed.

### Gaa^−/−^ mouse muscle retains its regenerative potential following acute injury

To determine whether the activation and myogenic commitment failure of the SCs was related to the absence of an activation signal in the Gaa^−/−^ mouse skeletal muscle, acute injury was performed with a CTX injection into the TA muscle of 4-mo-old Gaa^−/−^ and WT mice. This age was specifically chosen as it corresponds to the earliest stage during which SCs do not show signs of activation in response to disease-induced damage. The regeneration process was followed 4, 7 and 21 dpi.

**Fig. 9:**
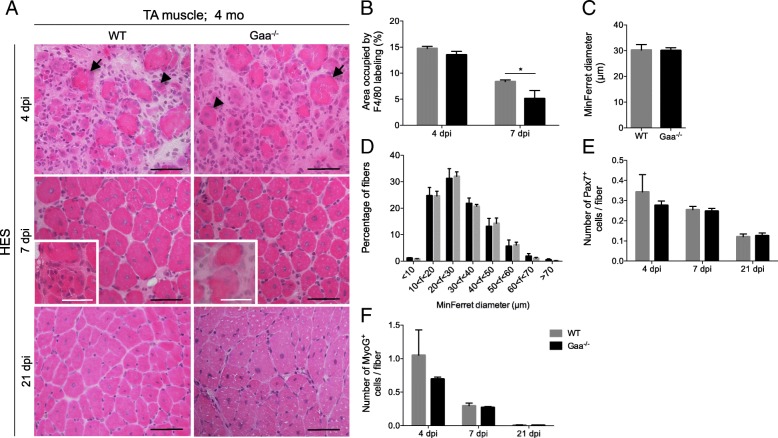
Similar muscle regenerative process in Gaa^−/−^ and WT mice following acute injury. **a**: Hematoxylin-Eosin-Saffron (HES) staining of *Tibialis anterior* (TA) muscle sections “Discussion”, 7 and 21 days after injury (dpi) induced by cardiotoxin (CTX) injection. Arrows and arrowheads indicate necrotic fibers and newly formed myofibers, respectively. Insert indicates necrotic fibers. **b**: Quantification of macrophage infiltration at 4 and 7 dpi based on F4/80 immunolabeling. **c**: Minimal Feret diameter of muscle fibers from Gaa^−/−^ and WT mice at 21 dpi. **d**: Distribution of TA muscle fibers from Gaa^−/−^ and WT mice based on their minimal Feret diameter. **e**: Number of satellite cells (Pax7^+^ cells) at 4, 7 and 21 dpi. F: Number of differentiated myoblasts (MyoG^+^ cells) at 4, 7 and 21 dpi. Scale bars = 100 μm. Statistics: Two-way ANOVA with Sidak post hoc test; n = 5 animals per group; *p < 0.05

At 4 dpi, the TA muscle from the Gaa^−/−^ mice was defined by scattered clusters of necrotic fibers with karyorrhectic nuclei as observed in the WT mice (Fig. 9a). Massive infiltration of both polynuclear and mononuclear inflammatory cells was observed. The area occupied by macrophages identified by the F4/80 immunolabeling was similar in the Gaa^−/−^ and WT mouse muscles, representing 14.75 ± 0.42% and 13.5 ± 0.69%, respectively (Fig. 9b). A few small centronucleated myofibers were also observed (Fig. 9a). At 7 dpi, large portions of necrotic debris still persisted with some polynuclear cells in the surrounding endomysium in the Gaa^−/−^ mice, while the WT mice displayed necrotic cytoplasmic debris that was progressively engulfed by numerous mononuclear cells (mainly F4/80 positive). The area occupied by macrophages appeared to be decreased by 1.63-fold in the Gaa^−/−^ mouse muscle compared with that in the WT mouse muscle (*p* < 0.05). The Gaa^−/−^ and WT mouse muscles exhibited active regeneration activity as determined by the numerous centronucleated fibers in the injured area. At 21 dpi, the Gaa^−/−^ and WT mouse muscles showed a similar degree of on-going tissue repair. The mean MinFeret diameter in the Gaa^−/−^ and WT mice was 30.10 ± 1.02 μm and 30.21 ± 2.18 μm, respectively, indicating no differences considering the global size of the muscle fibers (Fig. 9c). As shown in Fig. 9d, this finding was associated with a similar level of anisocytosis, and 91.70 ± 1.29% and 90.88 ± 3.69% of the fibers displayed a diameter ranging from 10 to 50 μm in the Gaa^−/−^ and WT mice, respectively. Between 4 and 21 dpi, the number of Pax7^+^ cells per fiber in the TA muscle similarly ranged from 0.28 ± 0.02 to 0.13 ± 0.01 and from 0.34 ± 0.09 to 0.12 ± 0.01 in the Gaa^−/−^ and WT mice, respectively (Fig. 9e). This finding revealed that the SC pool is not differentially impacted by the CTX-induced injury between the Gaa^−/−^ and WT mouse muscles. Concomitantly, the number of MyoG^+^ cells per fiber evolved similarly between the Gaa^−/−^ and WT mice (from 0.70 ± 0.03 to 0.01 ± 0.001 and from 1.05 ± 0.38 to 0.009 ± 0.002 between 4 and 21 dpi, respectively; Fig. 9f). This finding suggests that the TA muscles in the Gaa^−/−^ and WT mice share the same myogenic regulatory sequence. Collectively, the data generated by the in vivo muscle injury protocol showed that under an acute condition, SCs in this mouse model of Pompe disease are able to properly activate and efficiently contribute to muscle tissue repair.

### Loss of large fibers and splitting are typical features of aged Gaa^−/−^ mouse muscles

**Fig. 10:**
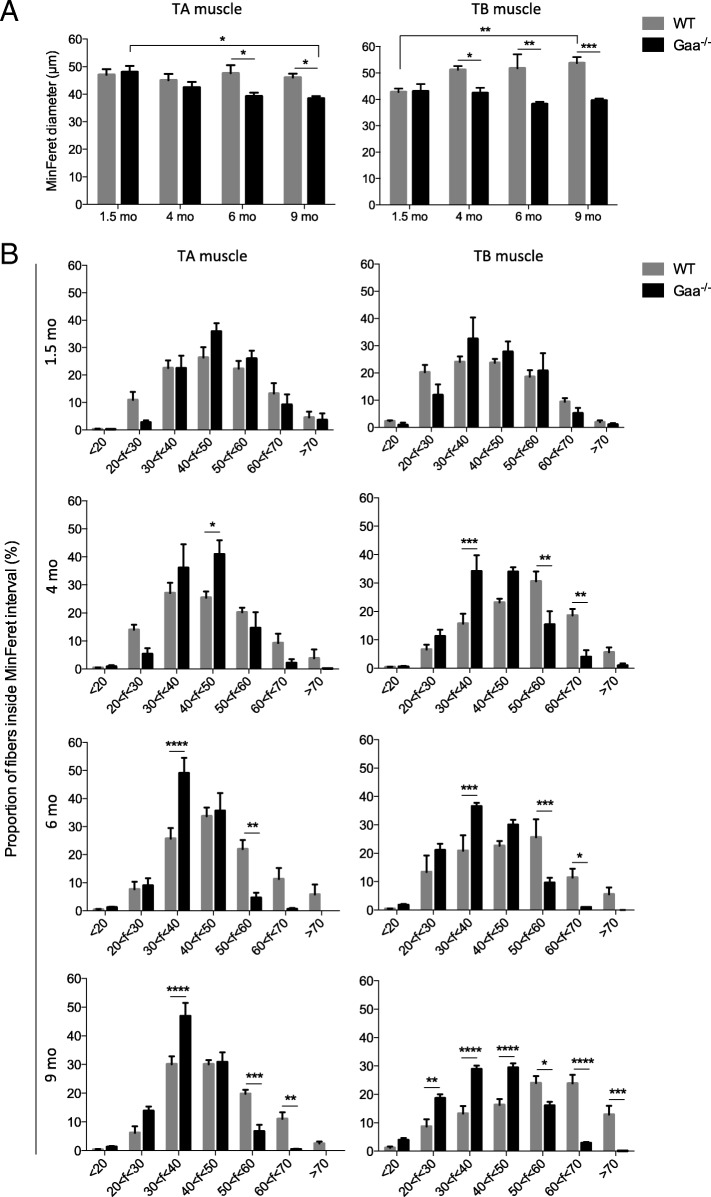
Anisocytosis in skeletal muscle fibers from Gaa^−/−^ and WT mice. **a**: Mean size of muscle fibers in *Tibialis anterior* (TA) and *Triceps brachii* (TB) muscles at 1.5, 4, 6 and 9 mo of age based on the minimum Feret (MinFeret) diameter. **b**: Distribution of muscle fibers according to the minimal Feret diameter in TA and TB muscles. Statistics: Two-way ANOVA with Sidak post hoc test; n = 5 animals per group; *p < 0.05; **p < 0.01; ***p < 0.001

Anisocytosis was measured to determine whether the fiber size in the TA and TB muscles sampled at the four time-points of this longitudinal study was altered in the Gaa^−/−^ mice considering the MinFeret diameter. In the TA muscle, the MinFeret diameter was 48 ± 2.24 μm and 42.4 ± 2.01 μm in the Gaa^−/−^ mice at 1.5 and 4 mo of age, respectively, whereas it was 47 ± 2.12 μm and 45 ± 2.39 μm in the WT mice, revealing no differences in the fiber size (Fig. 10a, left). Furthermore, the fiber size was consistently smaller at the two following time-points in the Gaa^−/−^ mice (39.2 ± 1.32 μm and 38.4 ± 0.93 μm) compared to that in the WT mice (47.6 ± 2.94 μm and 46 ± 1.41 μm). These results revealed that the fiber size significantly decreased over the course of the disease. Concerning the TB muscle, the mean MinFeret diameter of the fibers did not change or slightly decrease with aging in the Gaa^−/−^ mice. In comparison, an increased size was observed between 1.5 and 9 mo of age in the corresponding WT mice (*p* < 0.01; Fig. 10a, right). From 4 mo of age, a significant difference in the fiber size was observed between the Gaa^−/−^ and WT mice with an increased proportion of smaller fibers (*p* < 0.05). Interestingly, this loss of large fibers was not due to muscle atrophy since the mass of the TA and TB muscles in the Gaa^−/−^ mice did not differ from that in the WT littermates at each age considered. For example, the mass of the TB muscle relative to the weight of the mice corresponded to 0.37 ± 0.14% and 0.35 ± 0.01% in the 9-mo-old Gaa^−/−^ and WT mice, respectively. The distribution of the fibers according to their size at each time-point confirmed that no differences existed between the 1.5-mo-old Gaa^−/−^ and WT mice in the TA muscle (Fig. 10b). At 6 and 9 mo of age, the proportion of fibers exhibiting a MinFeret diameter greater than 50 μm was 5.22 ± 2.07% and 7.22 ± 2.39% in the Gaa^−/−^ mice compared with 38.94 ± 9.85% and 33.28 ± 4.24% in the WT mice, respectively (*p* < 0.0001). These results indicated a high decrease in the larger fibers at the advanced stages of the disease. Concerning the TB muscle, a similar fiber size distribution was observed at 1.5 mo of age between the Gaa^−/−^ and WT mice. From 4 mo of age, the proportion of fibers exhibiting a MinFeret diameter greater than 50 μm ranged from 10.59 ± 1.93% to 20.21 ± 7.65% and from 42.62 ± 8.85% to 60.70 ± 5.62% in the Gaa^−/−^ and WT mice respectively, revealing a severe reduction in the proportion of larger fibers in the Gaa^−/−^ mice (*p* < 0.0001). Overall, these results demonstrated a progressive decrease in fiber size over the course of Pompe disease that is characterized by the complete loss of the largest fibers (> 70 μm) and an enrichment of intermediate-sized fibers (ranging from 30 and 50 μm).

**Fig. 11:**
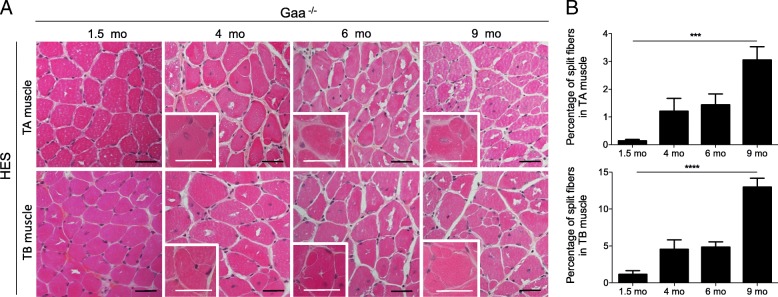
Splitting in skeletal muscle from Gaa^−/−^ mice aged 1.5, 4, 6 and 9 mo. **a**: Hematoxylin-Eosin-Saffron (HES) staining of *Tibialis anterior* (TA) and *Triceps brachii* (TB) muscles. Inserts show split fibers at a higher magnification. **b**: Quantification of split fibers in TA and TB muscles. Scale bars = 50 μm. Statistics: One-way ANOVA with Sidak post hoc test; n = 5 animals per group; ***p < 0.001; ****p < 0.0001

The analysis of the TA and TB muscles from the Gaa^−/−^ mice revealed the increasing presence of splitting that corresponded to the fragmentation of a fiber into two or more parts enclosed in a single endomysial tube, while no split fibers were observed regardless of the age considered in the WT mouse muscle (Fig. 11a). At 1.5 mo of age, both skeletal muscles displayed only rare and isolated splitting events (Fig. 11b). Furthermore, split fibers represented between 1.20 ± 0.42% and 3.05 ± 0.48% of all fibers in the TA muscle of Gaa^−/−^ mice aged between 4 and 9 mo. The proportion of split fibers in the TA muscle from the 9-mo-old Gaa^−/−^ mice was higher than that from the 1.5-mo-old mice with a 23.5-fold increase (*p* < 0.001). Similarly, between 4.53 ± 1.29% and 12.96 ± 1.22% of the fibers displayed splitting in the corresponding TB muscle. A 11.6-fold increase in the split fiber proportion was demonstrated in the TB muscle between 1.5 and 9 mo of age (*p* < 0.0001). From 4 mo of age, the TB muscle appeared much more affected than the TA muscle with a higher number of split fibers (*p* < 0.05 at 4 and 6 mo; *p* < 0.0001 at 9 mo). The splitting observed here resulted in an increase of the fiber number in both the TA and TB muscles of Gaa^−/−^ mice from the age of 6 mo (*p* < 0.0001).

## Discussion

Enzyme replacement therapy (ERT) with recombinant human GAA (Myozyme® and Lumizyme®, Sanofi Genzyme, Cambridge, MA, USA) can significantly increase the lifespan of patients with the infantile form by correcting the cardiac pathology [30, 48, 49]. Nevertheless, the response of skeletal muscle to ERT is highly variable among patients suffering from both forms of the disease. Indeed, although ERT improves motor and respiratory function in late-onset patients, skeletal muscle weakness persists, and some patients even show signs of disease progression [1, 2, 51, 66, 72, 78, 81]. The limited efficacy of ERT in treating the skeletal muscle impairment in Pompe disease highlights that the pathophysiology is incompletely understood. Consequently, a reconsideration of the muscle pathogenesis has emerged over the previous decade, highlighting that a dysregulation of the autophagy pathway is a hallmark of Pompe disease following the initial lysosomal glycogen accumulation due to GAA deficiency [37]. Several secondary disorders have been subsequently described, such as mitochondria defects, dysregulation of calcium homeostasis [36] or lipofuscin accumulation [12, 65]. The lack of muscle regeneration and a failure of SC activation has also been recently reported in patients [63]. Thus, better knowledge of the muscle pathophysiology underlying the mechanisms appears essential for proposing a more appropriate treatment. Here, we performed an extensive analysis of affected muscles in Gaa^−/−^ mice that recapitulate the features of both the infantile and late-onset form of the disease [54] because the progression of muscle lesions has never been exhaustively characterized. In particular, the phenotypic properties and functionality of SC have not been investigated.

Based on complementary approaches, we showed that an abnormally increased glycogen content was present from the age of 1.5 mo in the TA and TB muscles from the Gaa^−/−^ mice, confirming previous light microscopy data showing PAS^+^ material in the skeletal muscle of 1-mo-old Gaa^−/−^ mice [28, 61]. Importantly, we demonstrated that the glycogen content reached a saturated rate at this early age in both muscles and did not increase between 1.5 and 9 mo in the TA muscle and only slightly increased in the TB muscle at 9 mo of age. This finding is consistent with previous data from the *Quadriceps* and TB muscles of Gaa^−/−^ mice [89]. Furthermore, an increase in the glycogen content in *Quadriceps* muscle biopsies as the disease course progressed has also been reported in a few severely affected patients with the infantile form [77]. Interestingly, our data revealed that the original defect in the Gaa^−/−^ mice corresponding to the glycogen overload was quite disconnected from the intensity of muscle tissue remodeling characterized by increasing autophagic buildup, fiber splitting and centronucleation, which resulted in secondary consequences. These findings are concerning considering the progressive muscle function impairment occurring in Gaa^−/−^ mice over the disease course [11, 20, 28, 32, 52, 70, 89].

Autophagic buildup is the second hallmark occurring in Pompe skeletal muscle and has been reported in the late-onset form of the disease [55] and the infantile form among patients who survive longer with ERT [37, 58]. In the present work, autophagic vesicles were detected in the TA and TB muscles from Gaa^−/−^ mice aged only 1.5 mo, evoking a premature autophagic impairment in the murine model of Pompe disease. This notion is reinforced by work conducted by Fukuda et al. (2006), who demonstrated the presence of autophagic vesicles on isolated fibers in the *Extensor digitorum longus* muscle, TA muscle and *Gastrocnemius* muscle from 1-mo-old Gaa^−/−^ mice. We showed a cytoplasmic autophagic buildup over the disease course in both skeletal muscles characterized by a progressive increase in the number of fibers containing autophagic aggregates and the size of these aggregates. While the proportion of vacuolized fibers in the TA muscle from the Gaa^−/−^ mice was similar to that reported in adult-form biopsies [45, 65], this proportion appeared higher in the TB muscle, accounting for 50 to 69% of all fibers, but remained lower than that observed in biopsies from patients with the infantile form, where almost all muscle fibers appeared vacuolized [21, 45, 77]. In terms of the distribution inside the muscle fibers, the accumulation of autophagosomes was observed in the center of the cytoplasm, whereas the lysosomes were distributed uniformly, which is similar to previous descriptions of isolated fibers from 5- and 10-mo-old Gaa^−/−^ mouse muscle [17, 58].

Based on transmission electron microscopy investigations, several studies have shown that glycogen-filled lysosomes and autophagic buildup cause strong disruption of the myofiber organization with the loss of cross-striation [21, 69, 77, 87]. Notably, the present histopathological analysis of the TA and TB muscles showed very few fiber necrosis and weak macrophage infiltration, despite the substantial structural abnormalities. These data are consistent with findings reported in patient biopsies [63, 65, 77]. In addition, there were no extensive areas of fibrosis in both muscles, and none of the muscles studied showed fatty replacement. The same results were obtained in the *Quadriceps* and *Deltoid* muscles from late-onset Pompe patient biopsies [80]. Magnetic Resonance Imaging studies performed on patients suffering from the late-onset form have shown that muscle remodeling characterized by the development of adipose tissue exclusively occurred in the most severely affected patients [6]. Thus, Pompe disease is distinct from classical degenerative myopathies. A possible explanation for the absence of degenerative events despite the presence of ultrastructural damage may be that focal cytoplasmic injuries do not trigger sarcolemma disruption of Gaa^−/−^ muscle fibers.

We reported some differences in severity between the TA and TB muscles in terms of the percentage of fibers presenting autophagic buildup, centronucleated fibers and split fibers. This finding suggested that all skeletal muscles in Gaa^−/−^ mice may not evolve in the same manner. In contrast to patients, the accumulation of autophagic vacuoles in skeletal muscle has been shown to be limited to type II fibers in Gaa^−/−^ mice [53, 56, 57]. Nevertheless, the TA and TB muscles investigated here were almost exclusively composed of type II fibers as previously reported [23]. Muscle groups subjected to continuous or repetitive contractions were also shown to manifest greater histological damage in Pompe patients [80]. In addition, it would be interesting to further investigate these histopathological lesions in type I fiber-rich muscle, such as the *Soleus* muscle. Overall, these data support the notion that a differential pattern exists between skeletal muscles that needs to be considered, particularly in the context of the assessment of novel therapeutic strategies.

Regarding anisocytosis, we showed a loss of larger fibers in favor of intermediate-size fibers as the disease progressed in the two muscles considered. This finding is consistent with previous studies involving patients and Gaa^−/−^ mice [9, 45, 65, 86], although no increase in the smallest fibers was noticed in the present study. This result, combined with the observation of fiber splitting, represents a specific feature found among patients with other muscular dystrophies, myopathies or neurogenic disorders [67, 74]. To the best of our knowledge, this splitting has rarely been described in Pompe patient biopsies [65, 86], and no investigation of such splitting has been performed in Gaa^−/−^ mice to date. Several hypotheses have been proposed regarding the cause of fiber splitting. In some cases, fiber splitting seemed to be due to a biological process leading to the exclusion of necrotic areas of damaged fibers or subendomysial myoblast formation [64, 75]. Splitting has also been considered a compensatory phenomenon attributed to mechanical stress imposed on weakened muscles [4, 67, 75, 83]. In addition, it has been suggested that fibers split when a critical size is reached to maintain an efficient oxygen supply and metabolite exchange and a constant nucleocytoplasmic ratio [10, 74]. The relationship between mitochondria dysfunction during the aging process and fiber splitting has also been reported [24, 84]. Therefore, the mitochondrial defect reported in Pompe disease [36] could be in favor of an energetic defect to explain fiber splitting.

Interestingly, for the first time, we determined that the pool of SCs, whose participation is required for the regeneration of damaged fibers [33, 62], was preserved in both the TA and TB muscles regardless of the age considered between 1.5 and 9 mo. This result is consistent with that obtained in the *Quadriceps* muscle of patients with different disease severities [63]. Moreover, the proportions of activated SCs and myogenic committed SCs were detected in a similar range between the Gaa^−/−^ mice and WT mice from 4 mo of age, indicating that no SC activation occurred in response to disease-induced damage in adult skeletal muscle. Similar results were reported in muscle from Pompe patients, even the most severely affected patients [63]. One possible hypothesis explaining the failure of SC activation is that SCs are damaged in Pompe disease and unable to become activated. In support of this hypothesis, the proportions of activated and committed cells were found to be significantly higher in the 1.5-mo-old Gaa^−/−^ mice compared to those in the WT littermates. Since these Gaa^−/−^ mice exhibit a moderate skeletal muscle phenotype, it can be postulated that the secondary consequences of the glycogen overload are insufficient to prevent the activation of SCs at an early age. Moreover, the tissue injury induced by the CTX injection in the TA muscle of 4-mo-old Gaa^−/−^ and WT mice performed in the present study provided evidence that SCs retain their intrinsic potential to reconstitute muscle fibers following tissue damage, demonstrating that these SCs are clearly functional. This discordant behavior may be explained by the fact that the damage accumulated after the GAA defect, such as mitochondrial defect, dysregulation of calcium homeostasis [36, 58] or lipofuscin accumulation [12, 65], distorted the sensitivity of SCs to activate. It has been shown in the diaphragm muscle in a DMD mouse model that the accumulation of lipofuscin granules was associated with SC apoptosis as a result of oxidative stress [38, 44]. Although the SC pool remains intact in Pompe mice, lipofuscin accumulation and the subsequent oxidative stress could trigger defective SC activation and proliferation capacity. Thus, SCs could become active only in response to strong stimulation, explaining our results after the CTX injection. Indeed, such injections induce necrosis in all muscle fibers in the concerned area and disrupt the SC niche between the sarcolemma and the *basal lamina* [19]. To support this idea, it might be informative to subject Gaa^−/−^ mice to lower stimulation, such as training exercise, to determine how SCs exit their quiescence. In the present study, the SC activation was investigated in the Gaa^−/−^ and WT mice muscle. Nevertheless, a comparison with other low-grade smoldering disease processes like mild models of dysferlin deficiency would allow assessing to what extent there is a change in the baseline SC response specifically in Pompe disease and so enrich the conclusions.

Concomitantly to this defect of SC activation, we interestingly showed that the TA and TB muscles were characterized by the increasing presence of centronucleated fibers over the disease course, suggesting that a regenerative process occurred. However, very rare regenerated fibers were observed in these muscles. Such detection of centronucleated fibers was also described in previous studies performed in skeletal muscles from Gaa^−/−^ mice [73, 88, 89] and Pompe patients, in which a lack of regeneration have been demonstrated [59, 63, 65, 86]. Similarly, other muscle diseases have been described to present a high number of centronucleated fibers in the absence of the regeneration process. Notably, it is the case of the centronuclear myopathies that are due to mutations in gene encoding nuclear membrane proteins, which are known to contribute to nuclei movement through interaction with the cytoskeleton, specially microtubules [15, 29]. If nuclei positioned in the geometric center of the muscle fibers, which corresponds to a specific feature of centronuclear myopathies [60], are observed in the Gaa^−/−^ mice muscle, numerous nuclei exhibiting a random cytoplasmic location are also detected. Of note, a disorganization in the microtubular structure has been reported in regions exhibiting an overload of vesicles and autophagic debris in isolated fibers from Gaa^−/−^ mice [17]. Additionally, an impaired architecture of the cytoskeleton was highlighted over the course of the disease in an AGLU^−/−^ mouse model of Pompe disease [26]. Thus, a possible explanation for the presence of cytoplasmic nuclei with a non subsarcolemmal location in the Gaa^−/−^ mice may be an alteration in the cytoskeleton structure, impairing its repositioning under the sarcolemma. It would be informative to perform an in-depth exploration of the cytoskeleton structure over the course of the disease and investigate the relationship between myonuclei and proteins contributing to their movement.

Autophagy is known to be a key regulatory process in skeletal muscle homeostasis by playing a role in its development, growth and regeneration [22, 40]. Several muscle disorders, such muscular dystrophies [7, 13, 22, 50], lysosomal storage diseases [34, 68, 85] or vacuolar myopathies, are characterized by impaired autophagy [39]. Recent works have exhibited the role of autophagy in SC activation [13, 47, 76]. Indeed, the induction of autophagy has been shown to support the bioenergetic demands of quiescent SC activation. Furthermore, the inhibition of autophagy suppressed the increase in ATP levels and delayed SC activation [76]. Additionally, genetic impairment of autophagy in young SCs has been shown to cause entry into senescence due to the loss of proteostasis, increased mitochondrial dysfunction and oxidative stress [18]. Considering these findings and given the blockage of autophagic flux in Pompe disease skeletal muscle, an energetic defect could be the key element explaining the SC inactivation. Thus, it would be highly informative to investigate the energetic state of SCs to determine how autophagic impairment could be involved in their activation defect. Recently, the dysregulation of the mammalian target of rapamycin (mTOR) signaling has been described in skeletal muscle in Pompe disease [35]. mTOR is a serine/threonine kinase known as an energy and nutrient sensor [8]. Therefore, it could be interesting to explore the mTOR pathway precisely in SCs. Another known nutrient sensor that regulates autophagic flux in SC progeny is SIRT1, and its deficiency leads to delayed SC activation [76]. Thus, the role of SIRT1 in Pompe disease also deserves to be further explored.

## Conclusions

Our results demonstrate a lack of SC activation in adult Gaa^−/−^ mice that is maintained over the course of Pompe disease despite the increasing skeletal muscle damage. Our findings also provide evidence that SCs remain functional following acute injury, revealing a defect in the activation signal in Pompe disease. In addition, we identified fiber splitting, centronucleation and the loss of large fibers as typical histopathological signs that progress concomitantly with autophagic buildup as the disease progresses. Considering the growing demonstrations of the involvement of autophagy dysregulation in the pathogenesis, it could be informative to perform additional experiments to validate the hypothesis that the skeletal muscle tissue remodeling observed in the Gaa^−/−^ mice could result from a defect in the bioenergetic supply following impairment of the autophagic flux. The metabolic status of SCs over the course of Pompe disease should be explored.

## Abbreviations

BSA: Bovine serum albumin
CTX: Cardiotoxin
DMD: Duchenne Muscular Dystrophy
dMyHC: Developmental isoform of myosin heavy chain
dpi: Day post-injection
ERT: Enzyme replacement therapy
GAA: Acid alpha glucosidase
HES: Hematoxylin-eosin-saffron
IR: Infrared
MinFeret: Minimum of Feret diameter
mo: Month
mTOR: Mammalian target of rapamycin
MyoG: Myogenin
PAS: Periodic acid Schiff
PBS: Phosphate buffered saline
PCA: Principal component analysis
RT: Room temperature
SC: Satellite cell
SEM: Standard error of the mean
TA: *Tibialis anterior*
TB: *Triceps brachii*
WT: Wild-type
